# Barriers to home‐based palliative care in people with cancer: A qualitative study of the perspective of caregivers

**DOI:** 10.1002/nop2.503

**Published:** 2020-04-22

**Authors:** Hadi Hassankhani, Azad Rahmani, Amy Best, Fariba Taleghani, Zohreh Sanaat, Javad Dehghannezhad

**Affiliations:** ^1^ Center of Qualitative Studies School of Nursing and Midwifery Tabriz University of Medical Sciences Tabriz Iran; ^2^ Medical‐Surgical Department Nursing & Midwifery Faculty Tabriz Iran; ^3^ Medical Education Research Center Tabriz University of Medical Sciences Tabriz Iran; ^4^ Campus Teacher Acute Care School of Nursing Massey University Wellington New Zealand; ^5^ Nursing and Midwifery Care Research Center Faculty of Nursing and Midwifery Isfahan University of Medical Sciences Isfahan Iran; ^6^ Department of Hematology and Oncology Tabriz University of Medical Sciences Tabriz Iran; ^7^ Hematology and Oncology Research Center Tabriz University of Medical Sciences Tabriz Iran; ^8^ Nursing and Midwifery Faculty Tabriz University of Medical Sciences Tabriz Iran

**Keywords:** barriers, cancer, care, home, nurses, nursing, palliative

## Abstract

**Aim:**

To investigate the barriers to home‐based palliative care for cancer patients from professional caregivers' experiences.

**Design:**

A qualitative study.

**Method:**

This is a descriptive‐qualitative study carried out in the community‐based care. Twenty‐three participants took part in this study. Data were collected through semi‐structured interviews.

**Results:**

Data analysis led to the identification of three category of barriers including the lack of instructions (the lack of clinical practice guidelines, the ambiguity of tariffs and the lack of insurance coverage), family desperation (family views of prognosis, distrust and poverty) and lack of professionalism (limited knowledge, the use of amateur nurses and siloed care). Developing a care protocol and providing resources support contribute to the development of home‐based palliative care. Moreover, the education of families and training courses for nurses must be fostered.

## 1 INTRODUCTION

Cancer is the leading cause of death worldwide (Bray et al., 2018). Estimations suggest that in 2018, approximately 18.1 million new cancer cases and 9.6 million cancer deaths were recorded in the world (Ferlay et al., 2019). More than two‐thirds of reported cancer deaths occurred in low‐ to middle‐income countries. The number of new cancer diagnoses is increasing dramatically worldwide (Pilleron et al., 2019). By 2030, deaths from cancer are projected to reach over 13 million. Between “2008–2030,” in low‐ and middle‐income countries, the number of new cases of cancer is expected to increase by 80% (Bray, Jemal, Grey, Ferlay, & Forman, 2012).

Life‐limiting cancer has a profound effect on the individual and their family member's health and well‐being. This includes their physical, psychological, emotional, relational, social and spiritual health (McCaffrey, Bradley, Ratcliffe, & Currow, 2016). Quality of life (QoL) has also been shown to decrease significantly. Individual and family members support needs across the illness continuum therefore are dynamic, complex and multifaceted (Hui & Bruera, 2016).

The World Health Organization (WHO) has introduced palliative care as a solution for improving the life quality for patients with life‐limiting diseases and their families (WHO, definition of palliative care, 2018). It aims to improve QoL through the prevention and relief of suffering by assessing for and then treating the symptoms and psychological stress associated with terminal illness (Sewtz et al., 2018). Palliative care also includes assessment and support of primary caregivers. Palliative care can be initiated at any stage of the illness continuum. It is delivered in multiple settings such as the acute care setting, hospices and in the home. Therefore, multiple palliative care models exist that are context specific. Palliative care is an interdisciplinary specialty (Hassankhani, Rahmani, Taleghani, Sanaat, & Dehghannezhad, 2019).

Home‐based palliative care involves managing and caring for palliative patients in their home. There is a strong evidence base describing the benefit of home‐based palliative care in cancer patients. It is associated with improved patient and family outcomes, reduction in symptom burden and a more positive, satisfying experience for the patient and family. Patients are less likely to be admitted to hospital and those that are, have shorter lengths of stay (Gomes, Calanzani, Curiale, McCrone, & Higginson, 2013). Additionally, home‐based palliative care is associated with a reduction in use of healthcare services and resources (Lustbader et al., 2017) but the study by Shamieh and Jazieh (2010) revealed that the barriers to palliative care must be lifted to be able to provide the guidelines. In the Middle East states, these barriers are encountered in areas such as healthcare professionals, patients and families, manpower, drugs, education, funding and lack of national policies healthcare system.

## 2 BACKGROUND

There are currently no systematic structures for providing palliative care in Iran and only about 5–6 centres are active. These sites also provide palliative care in hospital and home care models. Several barriers to providing palliative care in Iran include the following: the lack of a defined structure for palliative care in the healthcare system, the lack of a defined job position for palliative care providers and the absence of serious training on palliative care in the formal curriculum of medical universities (Mojen, 2017); literature exploring cancer patient's wishes regarding end‐of‐life care shows that many have a preference for home‐based care. However, despite this, few cancer patients die at home; most end‐of‐life care is provided in hospital settings (Arnold, Finucane, & Oxenham, 2015). A study in Canada indicated that 70% of the participants preferred to die at home when they were about to die (Wilson, Cohen, Deliens, Hewitt, & Houttekier, 2013). Despite patient and family's desire for and interests in receiving home‐based palliative care, this service does not yet exist in Iran (Rassouli & Sajjadi, 2016). In response to these future predictions, the WHO recommend that all public health systems around the world give priority to the development and provision of palliative care services (Callaway, Connor, & Foley, 2018). In Iran, cancer is the third leading cause of death and the number of new cancer diagnoses continues to increase in line with global trends; 90,000 new diagnoses are made each year (Dolatkhah et al., 2015) and palliative care is in its infancy stage of development. The Ministry of Health have responded to calls for action from the WHO with advancements being made in (Rassouli & Sajjadi, 2016). Therefore, this study attempts to answer this question that what is barriers to home‐based palliative care in people with cancer of the perspective of professional caregivers?

This study seeks to identify and examine the barriers that prevent development of home‐based palliative care for cancer patients. Provision of good palliative care is a fundamental step to improving outcomes, the QoL and quality of care provided to terminal cancer patients and their families.

## 3 METHODS

### 3.1 Design

In this study, which has a descriptive‐qualitative design, samples were selected to analyse the viewpoints of the professional caregivers and transcend the barriers to home palliative care for cancer patients. We decided to carry out one‐on‐one interviews with the selected participants to gain a deeper insight. Data were collected from the one‐on‐one interviews, and data analysis and interpretation were carried out through the content analysis for the description of the qualitative data (Sandelowski, 1995).

### 3.2 Setting and participants

This study was directed between September 2018–April 2018 in the home care setting of East Azerbaijan Province, Iran. East Azerbaijan Province is a north‐western region of Iran. There are eight community‐based care in this province that provide home care to cancer patients. Four of these centres are in Tabriz City and the other four are in other counties. All these centres were selected to collect the samples for this study. A total of 23 participants, including eight managers of community‐based care, eight nurses with the experience of providing home care to cancer patients, an oncologist, a pain specialist, two nursing doctors and the nursing manager of the province, who were involved with home care, took part in this study (see Table 1 for demographic characteristics of all participants).

**TABLE 1:** Demographic characteristics of participants

Interview (*N* = 23)	
Gender	
Male	14
Female	9
Age bracket	
<19	0
20–39	11
40–59	12
>60	0
Position of participants	
Managers of community‐based care	8
Oncologist (MD)	1
A pain specialist	1
PHD of nursing	2
Bachelor of nursing	10
Nursing manager of the province	1
Job experience	
1–5 years	1
6–10 years	5
11–15 years	13
16–20 years	4

The inclusion criteria included the experience of working in community‐based care and the experience of caregiving or taking administrative actions for cancer patients. The participants were selected using the purposive sampling technique. Interviews were held with the interviewees who had a rich experience of providing home care to cancer patients. Besides, more information was collected from the managers, oncologists and nursing specialists. Maximum variety with a specific level of heterogeneity was considered as to age, work involvement and work environment type (urban or suburban) to test the members.

### 3.3 Data collection

Data collection was performed through in‐depth, semi‐structured and face‐to‐face interviews with 23 individuals from among the home care staff. They were initially provided with the necessary explanations. Participation in the study is voluntary and data will remain confidentiality and that they have the right to refrain from participation at any time without negative consequences. Once the informed consent was obtained, the interviews were conducted at the agreed time and place. The length of the interviews ranged between 30–45 min. The interviews were conducted by the first author and recorded by a digital recorder and then were transcribed down immediately after finishing the interview. To start the meeting procedure, some warm‐up enquiries were posed, for example, “what are your Education level?” and “how much experience do you have in home care settings?” Followed by the principle questions including:

- Describe your experience of providing home care to cancer patients.
- What are the barriers to the delivery of home palliative care from your point of view?

To enrich the collected data, the probing questions such as why, how, who and so on were asked during the interview. The interviews were continued until the time that no new data were obtained. As soon as the data repetition occurred, they were stopped (Speziale, Streubert, & Carpenter, 2011). In this study, saturation was reached when no new or contrasting results emerged from the interviews.

### 3.4 Data analysis

Analysis of the data was conducted using Lundman and Graneheim's (Graneheim & Lundman, 2004). Contractual content analysis process. The text of the interviews, after multiple reviews, was decomposed into the semantic units and then to the smallest meaningful units. Subsequently, the extracted codes were reviewed multiple times to be classified in the categories and subcategories based on their semantic similarity. After multiple reviews, the researcher and participants reached a semantic commonality about the categories. Furthermore, the researcher attempted to avoid considering his own presuppositions in data analysis. All the steps of encoding the text of the interviews were carried out in MAXQDA software. Analysis of the data was conducted by the primary author.

### 3.5 Trustworthiness

Data accuracy was validated using Graneheim and Lundman's model (Graneheim & Lundman, 2004). Four criteria are as follows credibility, dependability, transferability and confirmability. Data credibility was determined through constant engagement with the research subject, participants and data. Additionally, the text of interviews, extracted codes and subcategories were shared with some of the participants, whose feedback was considered. Dependability of the findings was demonstrated through accurate documentation and recording of all aspects of the research process which enabled a research process report to be written. Lastly, transferability of the data was demonstrated by sharing the findings with nurses who did not participate in the study but practised clinically in home care centres.

## 4 RESULTS

Eight nurses, eight cancer community‐based care managers, one provincial nursing manager, one oncologist, one pain specialist and two PhD nursing faculty members participated in the study. Interviews were conducted over 8 months and were of 30–45‐min duration. Findings from this study showed that three categorical barriers to home‐based palliative care existed**:** the lack of instructions, family desperation and lack of professionalism.

### 4.1 Lack of instructions

A lack of executive direction and clinical practice guidelines for the provision of home‐based palliative care of cancer patients emerged as a main barrier. This category is comprised of three subcategories: the lack of home‐based palliative care clinical practice guidelines, the ambiguity of nursing service tariffs and the lack of insurance coverage.

### 4.2 Lack of clinical practice guidelines

In Iran, palliative care services are in the early stage of development. There is currently no national framework for palliative care or clinical practice guidelines on home‐based palliative care provision. Community‐based care is common; however, even within these, there are no clinical guidelines on palliative care. Participants referred to failure of the Ministry of Health to develop such guidelines and protocols. One participant, who was the accreditation manager of a community‐based care, said:

When I analyzed all the directives and Instructions on home care, I did not find any instruction on the provision of home palliative care to cancer patients. Seemingly, providing care to these patients is carried out routinely and is limited to the caregiver's personal skills. (Manager 2)

### 4.3 Ambiguity in service tariffs

The participants referred to the lack of specific tariffs for home care services and the insufficiency of the supervision by the health system over these services. Moreover, they suggested that most centres charge the patients as they please and there is no single price for a given procedure:

When we were visiting the patients' houses, a discontent patient told me: Your center charges a lot because I had previously asked another center for the same service and it was cheaper. Each center charges a different price. I will take my patient to the hospital from now on because hospital services are cheaper. (Nurse 3)

### 4.4 Lack of insurance coverage

Participants unanimously agreed that there was an absence of financial support from state organizations for home‐based healthcare services. Additionally, insurance coverage was minimal for home care services. Participants stated that all costs are privately funded by patients. One of the participants who is the manager of a community‐based care stated:

One day a patient called the center and said…

I am a cancer patient. Since if have gastric cancer, I have to receive prescribed Total Parenteral Nutrition for a long time. If these services are provided at home, the insurance company will pay a percentage of the cost similar to hospital‐based services. (Manager 1)

According to the data, in addition to the shortcomings in the development of patient care guidelines, support for the patients' families is also neglected.

### 4.5 Family desperation

Regarding family's attitudes and perceptions of their loved one, participants stated that most families believed death was imminent and that nothing further could be done for the individual. They were not interested in the services of community‐based care due to financial cost/burden and distrust in services. However, if there was an absolute need for a nursing intervention to be delivered in the home, engagement with such services was inevitable. This category comprised of three subcategories: Family views of prognosis, distrust in healthcare services and poverty.

### 4.6 Family views of prognosis

Participants stated that a patient's family believe death is imminent once a cancer diagnosis is made. Because of this cultural belief that death is inevitable and fast approaching, they do not consider medical treatment or professional care appropriate. Most live on low incomes or in poverty and so the financial burden of care is too great. Central to this unwillingness to finance care is the cultural belief that the patient is already dead. One of the participants who is a nurse stated:

I went to the house of a patient suffering from leukemia and the patient's spouse asked me: When do you think our patient will die? Everybody says she will not last long and I am having financial problems. If she is not going to live, why should I spend this amount of money on her? (Nurse 1)

### 4.7 Distrust in healthcare services

Participants stated that cancer patients and families do not trust home care services. Instead, they place their trust in oncology clinics. Nurses that practice in oncology clinics and oncology hospitals provide home care to cancer patients:

I am a nurse with a vast experience of home care but in this area, the families of cancer patients mostly do not trust our home care nurses with nursing care. They prefer to use the clinic nurses or hospital nurses to provide home care to their patients. However, oncologists play a role because they tell their patients they have to stop visiting their clinics if they do not want the care provided by their nurses. (Nurse 2)

### 4.8 Poverty

Poverty appeared to be a significant barrier to cancer patients and families accessing home care services. Participants told how most cancer patients in Iran are in the low‐income class of society. They have often paid heavy expenses for medication and treatment and experience significant financial burden. As such, home care services for a dying loved one are simply not affordable. The lack of governmental support and insurance coverage for costs associated with home care services for cancer patients contributes to families not accessing such services. One of the nurses illustrates this when she says:

I visited a cancer patient at home. His family kept him in the parking lot due to financial issue. The patient received no health, social and spiritual care and he died in solitude. (Nursing doctor 1)

The resulting data suggest that the families of cancer patients are miserable and do not adequately trust home‐based care services. Meanwhile, the personnel of these centres provide care to cancer patients using the routine methods.

### 4.9 Lack of professionalism

Participants felt that a significant barrier to the development of home‐based palliative care services was a lack of specially trained healthcare personnel. They felt that nurses did not have enough theoretical knowledge on how to care for end‐of‐life cancer patients. They also felt that nurses did not have the clinical skills required to competently care for these patients and their families. Participants attributed this knowledge and skill deficit to a lack of education about palliative care at both the undergraduate university level, postgraduate level and in‐house education provided by clinical settings. This category consisted of three subcategories: limited knowledge, the use of amateur nurses and siloed care.

### 4.10 Limited knowledge

Of the participants who worked clinically, most expressed that they did not receive education or training in palliative care during their academic education. Furthermore, they stated that this lack of education continued in the clinical setting; community‐based care did not provide education on palliative care. They felt that educational workshops focused on the palliative care of cancer patients were required to be able to effectively care for these patients:

I am a nurse and I have been working for years in community‐based care for cancer patients. However, I have not taken any specific course on palliative care and I do not have adequate knowledge of this type of care. I think palliative care is synonymous with pain management. (Nurse 5)

### 4.11 Amateur nurses

Participants stated that bedside care in community‐based care was primarily provided by auxiliary nurses (auxiliary nurses are untrained care providers). Health care in Iran is privately funded. Registered Nurses are more costly for the health consumer than an untrained auxiliary nurse. Hence, given the lower cost of an auxiliary nurse, they are the preferred choice by families. However, a consequence of this is that the quality of care provided is lower when compared with that of a Registered Nurse. One manager said:

Many cancer patients cannot afford home‐based nursing care and they prefer auxiliary nurses. As a result, we cannot use register nurses more, hence the lower quality of the care provided to these patients. (Manager 4)

### 4.12 Siloed care

Another barrier to the development of palliative care described by participants was the siloed approach that currently exists; healthcare professionals from different disciplines work independently of one another. This siloed approach inhibits interprofessional communication and collaboration resulting in fractured care:

I am an oncologist and I am supporting a cancer patient at home. Meanwhile, I have realized that the psychologists, physiotherapists and nurses visit patients at their homes to provide home care. However, there is a lack of communication among us with regard to care provision and each one of us does her/his job separately and leaves. There is no teamwork or a center to make arrangements between the different sectors. (Oncologist)

The participants argued that integrated care is not provided to the patients and the care delivered is not high‐quality care because of an absence of communications between the caregivers and physicians. Therefore, the foundation of a centre that can make arrangements between different caregivers is a must and the lack of such a system hinders the growth of palliative care.

## 5 DISCUSSION

This study is one of very few that explore barriers to home‐based palliative care for cancer patients from the perspective of healthcare professionals working in community‐based care. Data analysis from this study has identified barriers to the development of home‐based palliative care services are the lack of clinical practice guidelines, family desperation and lack of professionalism at the point of care.

A key finding from this study is the lack of national instructions. There is no palliative care leadership at a national health system level. Such leadership could result in development of a strategic plan, clinical practice guidelines, protocols and care pathways. It is concluded that the lack of codes, which consists of the care protocol, nursing tariffs and insurance coverage subcategories, is one of the barriers to home‐based palliative care. This finding is in line with the findings reported by Bowman, Twohig, and Meier (2019), who described the ambiguity of the application of national standards to home care plans as one of the barriers to home‐based palliative care (Bowman et al., 2019). In a study in Germany, the barriers to home palliative care for children were the shortage of clear legal regulations and the lack of professional services (Jünger et al., 2010). Another study has introduced lack of legislation for palliative care integration as one of the obstacles (Zhi & Smith, 2015). In this regard, Kuwait Palliative Care Association managed to take steps in developing palliative care for cancer patients by taking measures such as encourage scientific research, inform the public, improve scientific knowledge, train volunteers in 2012 (Kuwait Palliative Care, 2020). Hence, management strategies explaining the national instructions and developing inclusive home palliative care plans and protocols in the health system are necessary.

The ambiguity of nursing service tariffs for cancer patients is another finding from this study, which fits into the category of “the lack of national instructions.” Similarly, Alppolito et al. (2016) reported that the clarity of nursing tariffs for home palliative care for each patient depending on the related complexities clarifies and identifies the care prices, thereby facilitating patients' access to the resources and the use of the resources (Ippolito, Boni, Cinque, Greco, & Salis, 2016).

Findings from this study show that lack of insurance coverage or financial constraints are a major barrier to the development of home‐based palliative care services for cancer patients. This is consistent with findings from Donkor's, Luckett, Aranda, and Phillips (2018) review which found that the financial limitation prevalent in low‐income countries was a primary barrier to palliative care provision (Donkor et al., 2018). Zhi and Smith (2015) assert that a crucial strategy in improving palliative care provision is increasing funding (Zhi & Smith, 2015). Greater financial support through more comprehensive insurance policies that cover costs associated with cancer care has also been shown to improve patients' outcomes (Soni, Sabik, Simon, & Sommers, 2018).

Therefore, the clarity of the tariffs of nursing services and financial support for home‐based palliative care protect cancer patients from confusion over the costs and enable their families to easily use these services and designation of nursing managers to develop a tariff‐based Nursing Services Implementation Plan and also financial support of patients will be helpful in developing palliative care at home for cancer patients.

Furthermore, the findings from the present study indicated the desperation of the patients' families is one of the barriers to the growth of home palliative care. This factor is classified into the following categories: family views of prognosis, distrust in services and family's poverty.

Family views of prognosis” is a finding that suggests the patient's family considered the patient to be as good as dead as soon as the cancer diagnosis was made. Once a diagnosis was made, family felt that treatment was “fruitless” and death was imminent. They were confused about the idea of receiving end‐of‐life care as they didn't see it would change the patient's outcome. Hurteau (2019) argues that misconceptions and a lack of knowledge about palliative care inhibit growth of the specialty (Hurteau, 2019). The lack of understanding on what palliative care is in Iran highlights the need for education to be provided around end‐of‐life symptom management. This finding supports Nemati, Rassouli, Ilkhani, & Baghestani, (2018) work identifying the challenges of families of cancer patients, uncertainty, confusion, setback and disintegration (Nemati, Rassouli, Ilkhani, & Baghestani, 2018). Zhi and Smith (2015) also cite family's confusion and agitation as major challenges to palliative care integration (Zhi & Smith, 2015). Hence, a paradigm shift is needed where patients and their loved ones in Iran understand the role of palliative care and what it can offer to both the patient and family in providing greater QoL and comfort at the end of life.

Families' lack of trust in home care services was another finding from this study. Ciemins, Brant, Kersten, Mullette, & Dickerson, (2015) work identified that families are satisfied with palliative care services if the individual care providers possess caring characteristics. The display of respect, empathy, compassion, genuineness, good communication and listening skills are important in establishing trust, rapport and service satisfaction (Ciemins, Brant, Kersten, Mullette, & Dickerson, 2015). By comparison, misconceptions and public lack of knowledge about palliative care services have been shown to restrict palliative care growth (Hurteau, 2019). Therefore, the establishment of a trusting relationship is essential to patient and family engagement with palliative care services and positive care experiences.

Another finding was the impact of poverty on access to services. Most families that the community‐based care cared for were impoverished and had severely limited access to financial resource. Insurance companies would not fund home care services. The lack of insurance cover leads to issues with paying for the care of a terminally ill family member which can be stressful and lead to financial hardship for the extended family. Yun et al. (2005) argue that the economic burden, which affects the life quality of cancer patients and their families, has to be lightened (Yun et al., 2005). A review study also revealed that cancer is a long‐term costly disease and a considerable number of cancer patients and their families are dealing with financial issues (Azzani, Roslani, & Su, 2015). Moreover, a study in Japan showed that the economic factor in the public community is one of the barriers to the growth of palliative care (Miyashita et al., 2007). Hence, it seems the identification of this issue by the health systems can contribute to the decrease in the damage inflicted on these patients by providing the necessary care and financial support.

The limited knowledge of home professional caregivers is another barrier to the development of home palliative care and a subset of the “lack of professionalism” class. The study by Mousing, Timm, Lomborg, and Kirkevold (2018) introduced limited knowledge as one of the barriers to home‐based palliative care (Mousing et al., 2018). In addition, a study in Europe revealed the shortage of training programs and limited knowledge as the barriers to palliative care (Lynch et al., 2010) and training is one of the palliative care development strategies recommended (van Riet Paap et al., 2015). Therefore, it seems holding training courses is effective in improving the knowledge of home palliative care and improving the quality of home care.

The use of auxiliary nurses instead of nurses reflects the unprofessionalness of home caregivers. A study in India confirmed that auxiliary nurses are employed in low‐ to average‐income countries due to the shortage of professional nurses for the provision of some types of care, which is a major concern regarding care quality. These auxiliary nurses need training (Rao et al., 2019). Additionally, another study highlighted the importance of nursing assistants' knowledge and skills in palliative care and recommended that nursing managers focus on increasing their knowledge and skills (Karacsony, Chang, Johnson, Good, & Edenborough, 2018). To enhance home care, preparation of a guideline for unregulated care providers has been recommended as well. Monitoring their performance is also helpful in developing and promoting home care (Saari et al., 2018). Thus, one of the management issues is to raise their knowledge and proficiency and to survey their performance and nursing managers had better take these elements into account.

The silo mentality and subsequent lack of interprofessional collaboration in cancer home care settings are another major barrier to the development of home‐based palliative care services. An integrated model of care centred on interprofessional collaboration where healthcare professionals work together to identify needs, make clinical decisions, deliver care and evaluate outcomes is considered essential for the provision of good palliative care. The study by Dudley, Ritchie, Rehm, Chapman, & Wallhagen, (2019) showed that when there is poor communication between care providers, the quality of care is poor as is the patient and family's experience of care (Dudley, et al. 2019). Patients' needs also fail to be identified and met. Therefore, an interprofessional collaborative approach to providing palliative care to patients and family's needs to be standard practice. In this study, most of the palliative care barriers were explored by the participants and it was not probe about the value of home‐based care in terms of quality of care that is one of the limitations of this study. And it is suggested to be considered in future studies. In this study, the barriers to palliative care were identified in the guidelines, patient's family and personnel areas. Therefore, a general guideline can be presented for the development of home‐based palliative care by offering solutions for removing these barriers.

## 6 IMPLICATIONS FOR NURSING MANAGEMENT

Data obtained from this study can serve as a starting point towards developing home‐based palliative care for cancer patients. Major barriers to providing good palliative care of cancer patients and home‐based palliative care in this context have been described. In response to this, strategies can be developed.

Education and training on palliative care are essential to the development of this service. Additionally, developing regulatory frameworks that govern practice and assess clinical competence of palliative care providers working in the home context is essential to facilitate safe practice and quality care. Findings from this study can also help policymakers develop clinical practice guidelines to inform palliative care provision in the home. A recommendation for further research would be exploring the barriers to home‐based palliative care from the viewpoint of patients.

Also, the findings from this study can also help policymakers develop instructions for the development of home palliative care for cancer patients, reduce the home care‐related concerns of cancer patients and take measures to improve the quality of the home care provided by nurses to cancer patients. Moreover, it is recommended to carry out a study on the barriers to home palliative care from the viewpoint of patients receiving home care to advance the knowledge of this approach.

## 7 CONCLUSION

Barriers for developing home‐based palliative care services can be classified into three levels: health system level; health consumer level; and healthcare professional level. At the health system level, there are a lack of instructions including a care protocol, tariffs of services and resource support.

At a health consumer level, patients and their family members lack understanding of palliative care, what it offers and do not trust home care services or the healthcare professionals at the point of care. To establish a specialist home‐based palliative care service for cancer patients, interventions need to focus on developing systems for funding of palliative care services, development of clinical guidelines and education of staff to build workforce confidence and capability in providing such specialist care. Recommendations include the following: development of a national framework and strategy for improving palliative care in Iran; development of evidence‐based clinical practice guidelines; palliative care education for all staff providing end‐of‐life care to ensure they are competent and up to date in the knowledge and practice that enables them to effectively care for and meet the complex, multidimensional needs of people living with a life‐limiting illness; and lastly inclusion of palliative care in undergraduate nursing curriculum. Finance is a challenge to advancing the provision of home‐based palliative care as health care is not publicly funded so this needs to be addressed at the political level.

## CONFLICTS OF INTEREST

All authors have no conflicts of interest.

## AUTHOR CONTRIBUTIONS

Hadi Hassankhani constructed an idea or hypothesis for research and/or manuscript; reviewed the literature; provided personnel, environmental and planning methodology to reach the conclusion; and supervised the course of the project or the article. Azad Rahmani involved in analysis and interpretation of data; took responsibility in logical interpretation and presentation of the results; and made critical revision. Fariba Taleghani planned methodology to reach the conclusion, and organized and supervised the course of the project or the article. Zohreh Sanaat reviewed the article before submission not only for spelling and grammar but also for its intellectual content; made critical revision and planning methodology to reach the conclusion. Javad dehghannezhad took responsibility in the construction of the whole or body of the manuscript; acquired the data; organized and supervised the course of the project or the article.

## ETHICAL APPROVAL

This study was approved by the Regional Research Ethic Committee at the Tabriz University of Medical Sciences (IR.TBZMED.REC.1397.675).
